# Personalized medicine: exploiting druggable vulnerabilities for KRAS-driven lung cancer

**DOI:** 10.18632/oncoscience.416

**Published:** 2018-06-23

**Authors:** Laetitia Seguin, Chloé Féral

**Affiliations:** INSERM, U1081, CNRS, UMR7284, Institute for Research on Cancer and Aging of Nice (IRCAN), University of Nice Sophia Antipolis, Nice, France

**Keywords:** lung cancer, KRAS, galectin-3, macropinocytosis, integrin

Non-small cell lung cancer (NSCLC) accounts for approximately 85% of all lung cancers and remains the leading cause of cancer-related death in the world [1]. Traditional cytotoxic chemotherapeutic agents have shown only very limited benefits for patients suffering from this type of cancer, highlighting the need for determining molecular drivers of NSCLC. Insights into molecular pathogenesis of lung cancers have identified mutations in specific genes involved in tumor initiation and progression. Among those, mutations in the Kirsten rat sarcoma (KRAS) oncogene represent one of the most prevalent genetic alterations in NSCLC. Not only KRAS mutant promotes stem like phenotype, cell proliferation, metabolic reprogramming and adaptation to the tumor microenvironment, it is also a marker of tumor aggressiveness and drug resistance [2, 3]. Therefore, KRAS has become the public enemy number one of oncologists and researchers, who have been working tirelessly to conceive therapeutic strategies aiming at interfering with its many functions. However, despite multiple approaches developed so far to interfere with mutant KRAS, its downstream signaling or its synthetic lethal vulnerabilities, and besides recent progress in targeting specifically KRAS G12C mutant, most strategies have failed in clinic due to compensatory mechanisms, alternative pathways, undesirable nonspecific effects, high drug toxicity and tumor heterogeneity. Indeed, one important obstacle in KRAS signaling inhibition strategies is the inter-and intra-tumor heterogeneity. One idea of personalized cancer therapy is to identify specific subgroups of cancer patients that will benefit from specific therapeutic strategies. Therefore, finding druggable target molecules to inhibit oncogenic KRAS signaling and identification of predictive biomarkers are key challenges in lung cancer therapy and more generally in cancer research.

Recently, identification of metabolic adjustments critical for KRAS-driven cancers has unveiled potential new axis for therapeutic intervention [4]. For example, macropinocytosis has been determined to function as a nutrient-scavenging pathway in KRAS-driven cancer cells. This clathrin-independent, actin-driven form of endocytosis involves the remodeling of the cell membrane to engulf large amounts of extracellular proteins and other macromolecules from the local microenvironment that will fuel different metabolic pathways, ultimately leading to the promotion of tumor cell survival and proliferation in adverse tumor microenvironment. These findings have highlighted macropinocytosis as a potential target for novel therapeutic strategies. Not only its inhibition represents a promising strategy to restrain tumor growth, but also its activation has emerged as a mechanism of entry for a variety of therapeutic agents including nanoparticles. Indeed, macropinocytosis-mediated Abraxane (Nab-paclitaxel) uptake represents a macrophage-activating strategy [5]. It is therefore critical to dissect the molecular pathways involved in this form of endocytosis in order to identify new molecular targets.

We recently described a previously unrecognized role of Integrin vβ3 as a functional partner of KRAS which, in cooperation with Galectin-3 (Gal-3), favors the survival of tumor cells by promoting macropinocytosis. While Integrin β3 is widely known as a cell-matrix adhesion molecule, which functions in adherent cells to integrate signals between the inside and the outside of the cell, we observed that in 3D primary lung cancer cell culture, integrin β3 is required for KRAS-mediated tumor cell survival and proliferation as well as downstream signaling. We found that Gal-3 promotes integrin β3 clustering independently of its matrix ligand (RGD)-binding domain, leading to KRAS recruitment and KRAS dependency [6]. Ultimately, this integrin β3/Gal-3/KRAS complex promotes KRAS-mediated macropinocytosis, allowing cancer cells to survive and proliferate in a hostile microenvironment. Moreover, the increase of reactive oxygen species (ROS) levels we observed upon pharmacologic inhibition of macropinocytosis indicates that this complex is involved in cell detoxification and also that macropinocytosis may be a KRAS-driven strategy to counteract redox imbalance [7].

It is now well established that cancer arises from deregulation of tissue repair mechanisms. Therefore, some insight into lung tissue repair may be gained from a consideration of the role of integrin β3/Gal-3/KRAS-mediated macropinocytosis in regenerative responses.

Finally, in a clinical perspective, we identified Gal-3 as a druggable vulnerability for a molecularly defined subgroup of lung cancer. Indeed, the use of GCS-100, a Gal-3 inhibitor already tested in clinical trial in patients with kidney and liver fibrosis, has shown specific cytotoxicity against β3-expressing lung cancer cells and regression of β3-positive tumors, by inhibiting the macropinocytic process and increasing redox imbalance [7]. Altogether, our study has unveiled a new therapeutic strategy but also identify a potential population of responders. However, the heterogeneity of β3 expression within the tumor, suggest that a combinatory strategy will be required to effectively target lung cancer.

As recent data have revealed a role for Gal-3 in the modulation of both innate and adaptive immunity, notably by inducing the apoptosis of T lymphocytes [8], and in light of our recent findings, one could envision that the combination of a Gal-3 inhibitor with immune checkpoint inhibitors might represent a potentially efficient anti-cancer treatment strategy. As a matter of fact, preclinical and early clinical trials combining Gal-3 inhibitor and PD-1 inhibitor have shown promising results for advanced melanoma, by reducing tumor burden and increasing survival (NCT02575404).

While we now appreciate that cancer is not just one but as many as patients, each with its own complexity, we understand that a single cure is not very realistic. Collectively, our results underscore the necessity to dissect cancer heterogeneity and to better understand KRAS-driven molecular mechanisms to stratify patients into progressively smaller subsets and rationalized combination therapies that will allow us one day to manage cancer as a chronic illness.
